# Covalent Organic Frameworks: A 2D Platform for Controlled Nanostructuring of Single Ion Magnets

**DOI:** 10.1063/4.0001129

**Published:** 2025-10-27

**Authors:** Noreen Mazhar, Mario Wriedt

**Affiliations:** The University of Texas at Dallas (UTD), Dallas, Texas, USA

## Abstract

Bulk magnetic materials play a crucial role in everyday life. Single molecule magnets (SMMs) and single ion magnets (SIMs), collectively known as molecular magnets (MMs), exhibit magnetic bistability and slow magnetization relaxation. Due to their large spin ground states and high axial magnetic anisotropies, MMs are gaining attention for potential molecular-level data storage and quantum computing applications. The discovery of the first known SMM, Mn12Ac, marked a breakthrough in magnetism for data storage devices. However, a significant challenge in molecular spintronics and MM design is their air sensitivity and functionality at ultralow blocking temperatures (TB < 10 K). While attaching MMs to surfaces like gold or graphene has been explored, this often results in a loss of symmetry and even destruction of their magnetic bistability. To enable reliable read/write functions, it is essential to organize MMs in a controlled manner that preserves their magnetic properties. In 2015, Wriedt et al. addressed this challenge by using 3D metal-organic frameworks (MOFs) as hosts for nanostructuring MMs. Building on this work, we aim to further explore MM behavior within 2D hosts such as covalent organic frameworks (COFs). We believe this approach could overcome many challenges related to encapsulating and separating individual MM domains. Ultimately, SIM@COF thin films have the potential for ultra-high data storage applications.

